# Editorial: Ice and Snow Algae

**DOI:** 10.3389/fpls.2022.868467

**Published:** 2022-03-14

**Authors:** Eric Maréchal, Linda Nedbalová

**Affiliations:** ^1^Laboratoire de Physiologie Cellulaire et Végétale, Centre National de la Recherche Scientifique, Commissariat à l'Energie Atomique et aux Energies Alternatives, Institut National de Recherche pour l'Agriculture, l'Alimentation et l'Environnement, Université Grenoble Alpes, Institut de Recherche Interdisciplinaire de Grenoble, CEA Grenoble, Grenoble, France; ^2^Department of Ecology, Faculty of Science, Charles University, Prague, Czechia

**Keywords:** snow alga, ice alga, *Sanguina*, red snow, bioalbedo, glacier, mountain

Natural environments covered with snow or ice are home to still poorly characterized microbial life. Photosynthetic organisms play a key role in colonizing these thermally labile habitats and creating conditions for complex communities to develop. The duration of these cold-adapted microbial communities can range from a few months to permanent settlements depending on the complete or partial melting of the snow and ice cover. In such habitats, algae do not only cope with low temperatures (psychrotolerance and psychrophily), but they can also be subjected to high and variable light levels, UV irradiance, low levels of nutrients (oligotrophy), and a variety of other abiotic stresses. In some cases, bloom-forming algae cause the formation of “green snow.” More often, they accumulate pigments, such as the carotenoid astaxanthin, leading to the development of “orange,” “pink,” or “red snow.” The abundance of pigmented microalgae lowers the albedo and accelerates melting. Algae are therefore both “markers” (positively impacted by current environmental changes) and “actors” (positively acting on ice and snowmelt) of climate change. Research on ice and snow algae is thus essential to better address the impact of climate change in polar and mountain environments.

Our knowledge of snow and ice algae is fragmented and relies on studies concentrated in some high latitude and high-elevation sites. Some algae are supposed to specifically propagate in the snow (“snow algae”) but we still have limited knowledge on the way they do. Their ecophysiological preferences and genetic diversity are still open questions. Some taxa seem to be dominant worldwide, in particular, the red snow alga *Sanguina* spp., detected in most high mountain ranges and polar areas, as in multiple studies reported in this Research Topic. An increasing number of algal species are being documented from the snow environment. Some are cultivable. In snow and glacier environments, green algae seem to be prominent, whereas diatoms, dinoflagellates, etc., are commonly encountered in sea ice. The present Research Topic compiles key contributions on the biodiversity, life cycles, (eco)physiology, developmental stages, and critical roles played by algae in ice (sea ice, glaciers, etc.) and snow, in high latitude polar and in high elevation mountain regions, in the context of climate change.

The determination of spatio-temporal distribution of taxa and the structure of communities rely on field sampling for the analysis of environmental DNA (eDNA), combined with other parameters, most notably chlorophyll and carotenoid pigments. Stewart et al. developed specific DNA-barcoding primers for Chlorophyta and Chlorophyceae, to analyze green algae biodiversity in top-soil along elevational gradients in five locations of the French Alps. They highlighted for the first time an altitudinal zonation of green algal taxa, some down to the genus level. The genus *Sanguina* was detected above the treeline, further assessing that this taxon is specific to alpine sites covered with snow for long periods and cannot be encountered at lower elevations in temperate regions. Environmental parameters and bioclimatic factors such as pH, C/N ratio, or intensity of freezing events proved determinant in algal distribution. Consistently, Williamson et al. analyzed the stoichiometry of macro-nutrients in the Southwestern margin of the Greenland Ice Sheet, revealing a low cellular macro-nutrient content and low C/N and C/P ratios, possibly reflecting adaptation of glacial algae assemblages to their specific oligotrophic surface ice environment. In Japan, Nakashima et al. addressed the structure of snow algal communities on Mt. Tateyama, combining eDNA with analyses of pigments. They could detect four general types of communities, some being dominated by *Sanguina* spp., whereas others were dominated by *Chlainomonas* and *Chloromonas* algae, causing different pigment compositions. Taking advantage of the relationship between pigment levels and snow algal biomass, Gray et al. used for the first time high-resolution WorldView multispectral satellite imagery to expand the scale of analysis of green and red snow blooms on Anchorage Island in the Antarctic. Future challenges rely therefore on the improvement of more specific eDNA markers for snow microbial communities, their use to unravel the spatiotemporal structure and dynamics of populations and communities in some study sites and correlation with satellite imagery-based approaches for more global characterizations.

Using strains isolated from sampling campaigns, refined taxonomic assessments can be achieved based on reconstructions of molecular phylogenies and analyses of cell morphological traits. If cultivable, ecophysiological studies can reveal some possible adaptation mechanisms. Galvez et al. thus identified a new Antarctic genus, named *Chlorominima*, with a species isolated in colored snow from the Collins glacier, therefore named *Chlorominima collina*. Together with other unidentified Antarctic and Arctic strains, a new polar subclade in the *Stephanosphaerinia* phylogroup within Chlamydomonadales is proposed. A partial transcriptome highlights the expression of genes coding for possible ice-binding proteins and enzymes involved in the synthesis of triacylglycerols and carotenoids, which could play a role in the adaptation to a cold environment. By contrast, Morales-Sanchez et al. showed that the polar alga *Chlamydomonas malina* RCC2488 did not accumulate triacylglycerol at low temperature (4°C) but rather at higher temperatures (8°C and above), suggesting that for this species, triacylglycerol may not be an adaptation to low temperature but a response to a high-temperature stress. Raymond et al. showed that two psychrophilic *Chlamydomonas* species isolated from Lake Bonney, a saline lake in the Antarctic, synthesize glycerol, an osmoprotectant, in a NaCl-dependent manner. Glycerol is synthesized by an unusual bidomain enzyme previously characterized in *Chlamydomonas reinhardtii*. In one of the Antarctic strains, they could identify an isoform of this enzyme, which expression is controlled by NaCl level. Eventually, Procházková et al. isolated a strain causing orange snow in the High Tatra Mountains in Poland and identified it as *Chloromonas krienitzii*, a species previously described from Japan. They characterized a unique mechanism shielding its algal cysts from a substantial part of UV irradiance and high visible light, by the presence of short wavelength-absorbing compounds in the cell wall. These articles illustrate the diversity of adaptation mechanisms that snow and ice algae may develop to live in their peculiar habitats. Factors determining the capacity to form blooms are still unknown. Future studies are therefore needed to evaluate which of these mechanisms are generic, shared by multiple species distant in the evolution, which are more specific to some taxa, and how these mechanisms may be related to the capacity to form blooms.

Eventually, considering that for tens of millions of years (720-635 Ma before present), the terrestrial habitats of the so-called “Snowball Earth” were likely dominated by snow and ice, Žárský et al. develops a scenario for the Zygnematophyceae–Embryophyta split, possibly stimulated by this long glacial period.

Altogether, the contributions to this Research Topic illustrate the dynamic international efforts to fill gaps in knowledge on algae living in the snow and ice. The efforts to characterize populations and communities in high elevations above treeline and polar areas, their dynamics, functioning, connectivity with other habitats such as soil, rivers, lakes, and oceans, together with genomic, ecophysiology, and multi-omics studies are likely to help us unravel adaptation mechanisms and to understand what it really means to live in the snow and the ice. Fascinating studies are expected in the near future.

## Author Contributions

Both authors listed have made a substantial, direct, and intellectual contribution to the work and approved it for publication.

## Funding

EM is supported by the French National Research Agency (BLinK ANR-18-CE92-0015, Alpalga ANR-20-CE02-0020, GlycoAlps ANR-15-IDEX-02, GRAL Labex ANR-10-LABEX-04, and EUR CBS ANR-17-EURE-0003), Institut Carnot 3BCAR and the Kilian Jornet Foundation.

## Conflict of Interest

The authors declare that the research was conducted in the absence of any commercial or financial relationships that could be construed as a potential conflict of interest.

## Publisher's Note

All claims expressed in this article are solely those of the authors and do not necessarily represent those of their affiliated organizations, or those of the publisher, the editors and the reviewers. Any product that may be evaluated in this article, or claim that may be made by its manufacturer, is not guaranteed or endorsed by the publisher.

